# The Effect of Celecoxib on the Progression of Calcific Aortic Valve Disease—Protective or Pathogenic?

**DOI:** 10.3390/jcm12072717

**Published:** 2023-04-05

**Authors:** Zachary Vinton, Kevin Wolfe, Jensen Fisher, Amanda Brooks

**Affiliations:** 1 College of Osteopathic Medicine, Rocky Vista University, Parker, CO 80112, USA; 2 Department of Library Services, Rocky Vista University, Parker, CO 80112, USA; 3 Office of Research and Scholarly Activity, Rocky Vista University, Parker, CO 80112, USA

**Keywords:** celecoxib, NSAIDs, COX-2, calcific aortic valve disease, aortic stenosis

## Abstract

Calcific aortic valve disease (CAVD) is a debilitating condition for which there are limited therapeutic options aside from valve replacement. As such, it is crucial to explore alternative management strategies for CAVD. Non-steroidal anti-inflammatory drugs (NSAIDs), particularly celecoxib, have been the subject of debate in the literature regarding their potential impact on CAVD. We conducted an in-depth analysis of five studies exploring the effect of celecoxib on CAVD and found discrepancies in both methods and results. Our findings suggest that celecoxib may impact the development of this disease via multiple mechanisms, each of which may have different effects on its pathogenesis. We also discovered limited clinical research examining the connection between celecoxib use and CAVD in medical patients. As such, further studies are needed to clarify the role of celecoxib and other NSAIDs in CAVD progression in order to inform future treatment options and clarify their impact on the disease.

## 1. Introduction

Calcific aortic valve disease (CAVD) is accompanied by mineralization of bicuspid (half of all removed calcified valves are bicuspid) [1] or tricuspid aortic valve leaflets, leading to a progressive decline in function of the aortic valve via both decreased valvular area and increased valvular narrowing, resulting in a reduced blood flow through the leaflets [2]. Between 1990 and 2019, the global incidence of CAVD has increased by a factor of 3.51 (589,000). The prevalence has increased by 4.43 (9,404,000) and attributable deaths have increased by 1.38 (126,000), making it the most common valvular disorder and a significant cause of morbidity and mortality worldwide [3]. Due to systemic ramifications such as sudden death (in severe aortic stenosis), heart failure, pulmonary hypertension, infective endocarditis (particularly in patients with bicuspid valve calcification), bleeding, systemic emboli, and strokes [4], it is the most common indication for surgical valve replacement [5]. In fact, the only treatment modality currently available is surgery, which emphasizes the importance of uncovering new interventions as well as further illuminating the disease process behind CAVD.

## 2. Pathogenesis of CAVD

**Figure 1:**
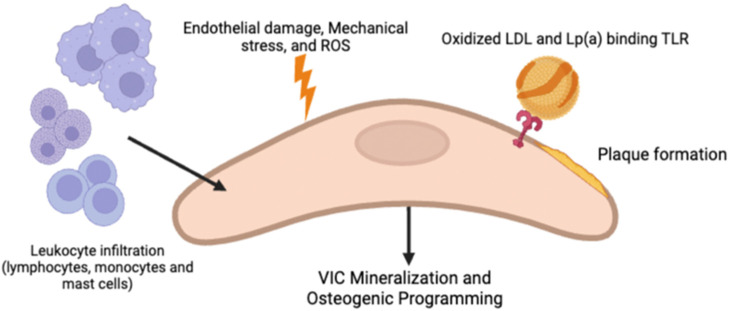
Pathogenesis of CAVD involves lesions from endothelial damage, mechanical stress, and ROS. This allows for oxidized LDL and Lp(a) infiltration, plaque formation, and subsequent leukocytic infiltration of VICs. This leads to progressive mineralization and osteogenic programming of VICs [4,6].

Previous hypotheses of the pathogenesis of CAVD included passive calcification and normal degeneration of the aortic valve; however, it has been found to be more complicated. Current understanding of the disease process includes chronic inflammation, lipoprotein deposition, and active leaflet calcification [6] contributing to progressive calcification. As shown in Figure 1, valvular interstitial cells (VIC) are the predominant aortic valvular cells and under normal circumstances are thought to reinforce valvular structure; however, they are suspected to be one of the disease drivers in CAVD, as they acquire pro-calcific characteristics due to pathological stimuli such as lipoprotein accumulation, endothelial damage, inflammatory mediators, reactive oxygen species (ROS), increased calcium and phosphate levels, and cyclic stretch [4,6]. 

## 3. Inflammation

Similar to atherosclerosis, repeated endothelial damage is thought to be responsible for triggering the development of CAVD due to the loss of valvular homeostasis via reduced shear stress and increased mechanical stress. Subsequently, vascular cell adhesion molecule-1 (VCAM-1) and intracellular adhesion molecule-1 (ICAM-1), which are hallmarks of early stages of inflammation, are upregulated [1,7]. These cell signaling cascades lead to inflammatory cells (macrophages, T-Lymphocytes, and mast cells) being recruited into leaflets and the infiltration of lipoproteins (LDL and Lp(a)), forming subendothelial plaque-like lesions resulting from LDL oxidation due to the release of ROS from the inflammatory cells [1]. The binding of oxidized lipid species to TLRs on the VICs, as well as the activation of the NF-KB pathway via TNF (secreted by immune infiltrates such as macrophages and monocytes) and binding to TNFR1, are both thought to promote VIC mineralization and osteogenic programming [2]; however, how the pro-calcific VIC causes ECM mineralization is still not fully understood. In addition to osteogenesis, it is also postulated that apoptosis of VICs via ROS, cytokines, and purinergic signaling may lead to dystrophic calcification containing calcium and phosphorous crystals in CAVD [2]. Neovascularization often accompanies inflammation. Although the mechanism of its involvement in CAVD is not entirely clear, it is postulated that it is involved in recruiting both inflammatory cells and osteoprogenitor cells.

## 4. NSAIDs

**Figure 2:**
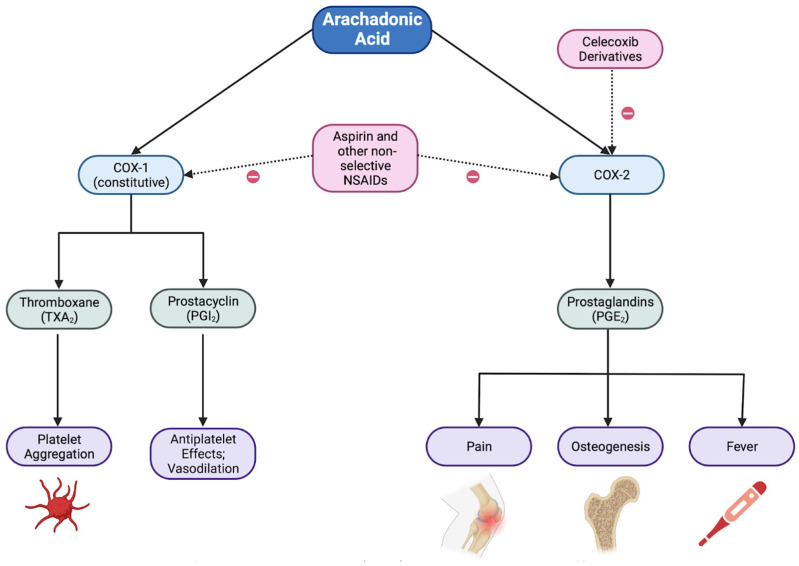
A summary of the cyclooxygenase (COX) pathways and their effects. Celecoxib and its derivatives selectively block the COX-2 pathway, inhibiting pain, inflammation, and fever without impacting platelet aggregation [7,8].

The COX-1 and COX-2 pathways are responsible for converting arachidonic acid to products that mediate pain and inflammation, as shown in Figure 2 [8]. COX-1 produces thromboxane A2, and both COX-1 and COX-2 are responsible for the production of prostaglandins [9]. The COX-2 pathway, specifically, produces prostaglandins during inflammation [9]. COX inhibitors are a class of non-steroidal anti-inflammatory drugs (NSAIDs) that are used to block the COX-1 and COX-2 pathways to reduce pain, inflammation, and fever [8]. However, some COX inhibitors, such as COX-2 inhibitors (the majority of which have been discontinued from use) and aspirin, are associated with cardiovascular side effects [10]. It is still unknown whether COX inhibitors might play a role in the development of CAVD; however, studies have attempted to identify whether there is a connection due to the role these drugs play in inflammation and cardiovascular risk [10].

## 5. Mineralization

It is suspected that the cytokine IL-6, a central regulator in chronic and other immune-mediated responses plays a role in CAVD through its involvement in increasing the expression of NF-KB.2. IL-6 increased in human calcified stenotic valves, likely due to the expression of RANKL (receptor activator of an NF-KB ligand), which thereby activates RANK. RANKL causes VICs to increase the production of the extracellular matrix (ECM) [11]. Nucleation of calcium and phosphorus can begin on this secreted ECM. IL-6 also promotes mineralization through the BMP2 pathway [2]. Interestingly, Weiss et al. demonstrated that Osteoprotegerin administration, which is a decoy of RANKL, attenuated calcification of the aortic valve in mice and preserved valvular function [12]. The role of various proteins present in the ECM, such as proteoglycans and periostin, are thought to be involved in the remodeling of the aortic valve during aortic stenosis (AS), but this is not yet fully understood. For instance, osteopontin and bone sialoprotein are drastically upregulated at sites of calcification and help attach osteoblasts to bone matrix [13].

Other cytokines that may be involved are IL-1B and IL-1, which increase the expression of matrix metalloproteinases (MMPs). These enzymes degrade ECM, exacerbate stenosis, and activate the NF-KB pathway, leading to an increase in IL-6, IL-8, and MCP-1. IL-37, which attenuates bone morphogenic protein (BMP2) and alkaline phosphatase, both of which inhibit osteogenesis, is in the same family as IL-1B. In patients with CAVD, levels of IL-37 are low, leading to BMP2 promoting the thickening of the aortic valve [1]. Beyond the BMP pathways, both the angiotensin-converting enzyme (ACE) and chymase increased in CAVD. Chymase (via mast cells) and ACE both convert angiotensin I into angiotensin II. Angiotensin II (with a type AT1 receptor found in CAVD) [13] correlates with TNF and IL-6 expression and is pro-fibrotic [14], making it an important aspect in the pathogenesis of CAVD. In hypercholesterolemic rabbit models, it was found that angiotensin receptor-1 blockers (ARBs) were capable of preserving the endothelial integrity of the aortic valve while disrupting transdifferentiation into osteoblasts and/or myofibroblasts [15].

Additional factors thought to contribute to CAVD are genetic predispositions. Bicuspid valves, which are susceptible to calcification, are associated with NOTCH1 mutations. Normally, NOTCH1 in VICs helps to prevent the expression of BMP2 and RUNX2, which are osteogenic factors, meaning that some patients may be genetically susceptible to developing CAVD. Moreover, the WNT pathways in patients with CAVD are overexpressed, which may also lead to calcification. The above factors contribute to the fibrosis and calcification of the aortic valve, ultimately leading to sclerosis and the necessity for surgical valve replacement. More research is necessary to illuminate the complexities behind the disease processes of CAVD.

## 6. Treatment Options

Currently, there are no treatments available for calcific aortic valve disease aside from surgical aortic valve replacement (SAVR) and transcatheter aortic valve implantation (TAVI/TAVR) (for patients with increased operative risk) [16]. This is problematic because of the risk for complications, including endocarditis and thrombosis, along with a limited valve lifespan, often leads to reoperation [4].

Based on the ACC/AHA Guideline for the Management of Patients with Valvular Heart Disease, an intervention for calcific aortic stenosis is only indicated if (1) CAVD is severe, (2) the patient has a life expectancy greater than one year with surgery, and (3) the intervention is likely to improve the patient’s quality of life [17]. This is evaluated with a multidisciplinary heart valve team involving a cardiologist with expertise in structural valve intervention and a cardiothoracic surgeon. Indications for SAVR over TAVI include another indication for cardiac surgery (CABG or mitral valve surgery), patient age under 75, characteristics indicating a mechanical valve replacement (can only be placed surgically), or anatomic features increasing the risk of TAVI complications, such as adverse aortic root or a severely calcified bicuspid valve. If SAVR is not indicated, the transfemoral TAVI is a choice with a robustly lower hazard ratio and mortality. Notably, mortality was not reduced with transthoracic TAVI in comparison with SAVR and transfemoral TAVI. Indications for TAVI over SAVR include a patient aged 75 or higher, high feasibility of transfemoral TAVI, risk factors for SAVR (frailty or cirrhosis), and the female sex (lower mortality under TAVI compared to women w/SAVR). Risks for SAVR are evaluated using the STS risk estimate, frailty, major organ system dysfunction, and procedure-specific impediments. Patients are at intermediate risk, which is classified by an STS of 4–8%, when at least one indicator of frailty is present.

There are currently no drugs available for the treatment or prevention of aortic stenosis. Celecoxib, a selective COX-2 inhibitor, has been investigated as a potential solution to this gap in pharmacotherapeutic interventions [18]. There are two mechanisms by which the drug is proposed to prevent the progression of calcification of the aortic valve; however, at the time of writing this review, there has been limited research into this topic [18,19].

## 7. Materials and Methods

A comprehensive review of academic publications was performed to answer the following question: is there a connection between the use of celecoxib and the development of calcific aortic valve disease? An assessment of our current knowledge of this topic was accomplished by conducting a broad search of the literature, selecting relevant articles, and synthesizing the findings from each study in order to develop a uniform picture of our current understanding. The literature search was conducted in PubMed, Cochrane, Google Scholar, and ClinicalTrials.gov using the keywords NSAIDs, COX-2, calcific aortic valve disease, aortic stenosis, and celecoxib. Inclusion criteria consisted of studies that examined the possible connection between either the COX-2 pathway or celecoxib and the development of aortic valve calcification (AVC). Studies in both humans and animals that utilized quantitative data were accepted. Due to the focus of our review being on a topic that has not been studied robustly, further inclusion criteria were not incorporated. For the purpose of answering this question with up-to-date statistical information, exclusion criteria included studies that were published more than ten years ago and studies that utilized qualitative methodology.

## 8. Results

Due to the role of the COX pathways in inflammation and the known connection between NSAIDs and cardiovascular events [20], there has been an investigation as to whether the COX-2 pathway has a role in calcific aortic valve disease (CAVD). The investigation of this report identified five relevant studies, shown in Table 1, to help address this question and found that there is no current consensus in the literature regarding the connection between the two. Some studies suggest that upregulation of the COX-2 pathway could potentially be disease-driving in AVC [18]. However, others have suggested the opposite; that COX-2 has a protective effect against it. Therefore, COX-2 inhibitors could play a role in worsening the development of AVC and subsequent aortic stenosis [21]. Other studies have suggested that there may not be an association between COX-2 inhibitor use and AVC at all [22].

One study found that the COX-2 pathway had increased expression in human calcified aortic valves [18]. That finding alone, however, does not necessarily mean that COX-2 drives calcification. More clarification is needed to determine whether COX-2 upregulation is driving calcific disease, or whether it means that upregulation is a protective response to another disease process causing the calcification. However, inhibition of the COX-2 pathway with celecoxib also reduced the induction of calcification in a mouse model, supporting the idea of a cause-and-effect relationship in which COX-2 activity leads to AVC. At a glance, this fits well considering COX-2 has a known role in bone healing [24], and these findings would suggest that celecoxib or other NSAIDs could potentially serve as therapeutics for AVC prevention.

However, another in vitro study performed on human aortic valve leaflets sampled from patients with aortic stenosis directly contradicts these findings [21]. In this study, it was found that the COX-2 pathway actually had decreased expression in calcified valves. The addition of a COX-2 inhibitor, celecoxib, to these samples also induced further calcification. This would lend credence to the idea that the COX-2 inflammatory pathway has a protective effect, and its downregulation allows for calcification and subsequent stenosis to occur. As a result, it could be assumed that celecoxib and other COX inhibitors are risk factors for the development of aortic stenosis and would be contraindicated in patients at risk for it. As the primary cause of aortic stenosis is age-related calcification, this could be a major contraindication to a class of drugs already not widely used due to associations with other cardiovascular events (although celecoxib is proposed to be the safest in this regard) [25]. These findings are in direct opposition to those of Wirrig et al. (2015), although this difference was suggested to potentially be due to differences in the methods of measuring [18,21]. At the very least, however, the contradictory findings suggest that more investigation is needed to clarify this association.

There were also different proposed mechanisms regarding how celecoxib could affect the development of CAVD, which may help explain the contradictory findings. Celecoxib is proposed to have another non-COX-2-associated effect that could potentially be protective against AVC, through a CDH11 blockade [19]. The CDH11 transmembrane protein has been found to have increased expression in calcified aortic valves, serving as a potential therapeutic target [26]. Celecoxib and its derivatives have been shown to have a high binding affinity for this protein. Both celecoxib and dimethyl celecoxib (DMC), a celecoxib derivative with action against CDH11 but no inhibitory effects on COX-2, were investigated as potential therapeutics for the treatment of aortic stenosis. However, in one in vitro study, celecoxib was shown to actually be associated with increased calcification, further supporting the idea that COX-2 inhibition may be pathogenic, while DMC had the expected protective effect against calcification [19]. Vieceli Dalla Sega et al. (2020) agrees with these findings with regard to celecoxib, which again are in direct contradiction to Wirrig et al. (2015) [18,21].

More recently, another study performed a number of experiments with different conditions and variable findings that may explain the controversy as to whether the COX-2 inhibitors celecoxib and DMC are protective or pathogenic in the development of AVC [23]. Like previous studies, the authors replicated the potential increased risk for AVC in an in vitro environment using explanted porcine aortic valve leaflets in osteogenic media. This finding was observed with both celecoxib and DMC, suggesting that this potential pathogenic effect is not characteristic of COX-2 inhibition. However, this effect was not observed in studies that were performed without dexamethasone, suggesting that the pathogenic effect of celecoxib and DMC may be due to yet another non-COX-2-associated effect that is dependent on the presence of glucocorticoids. With dexamethasone removed from the osteogenic media, the effect was reversed as expected. Furthermore, the authors found that co-treatment with a MEK1/2 inhibitor rescued this pathogenic effect, suggesting an involvement of the MEK/ERK pathway in this glucocorticoid-dependent effect. The findings of this study, particularly the suggestion of another potential mechanism of action of celecoxib, may explain the previous controversy as to whether COX-2 inhibition is protective or pathogenic in the development of AVC if the reason for the contradictory findings in previous studies was the presence of glucocorticoids in in vitro media that were used.

These findings suggest both an explanation for the pathogenic effect of celecoxib and its derivatives, as well as the potential for therapeutic prevention of AVC, if these non-COX-2 effects can be further studied and understood. If a glucocorticoid-dependent effect causes the administration of celecoxib and its derivatives to drive calcification of the aortic valve, then it would suggest the need for studies of whether this effect is present in in vivo conditions when the drug is administered. It also suggests that if this pathway could be eliminated, such as with an MEK 1/2 inhibitor, celecoxib and DMC may still serve as potential therapeutics through the blockage of either COX-2 or CDH11. This highlights the need for further studies and suggests that celecoxib and its derivatives could either be potential therapeutics or disease-driving agents in CAVD and consequent aortic stenosis.

**Figure 3:**
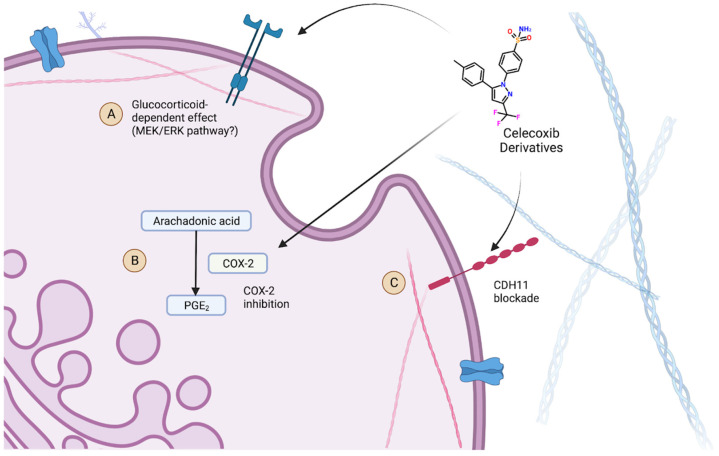
A summary of the pathways celecoxib and its derivatives that are proposed to affect (**A**) the glucocorticoid-dependent effect (possibly MEK/ERK), (**B**) the inhibition of COX-2, and (**C**) the blockage of the CDH11 transmembrane protein [9,19,23].

The impact of celecoxib and its derivatives on the development of AVC remains unclear with our current breadth of knowledge. However, there is evidence to suggest that celecoxib can affect three cellular pathways as shown in Figure 3, including the inhibition of COX2, CHD11 blockades, and potentially a third glucocorticoid-dependent effect. While there is debate as to whether COX2 inhibition can either promote or prevent AVC, it seems more certain that the CDH11 effect does help prevent its development. Conversely, it can be concluded that their effect in the presence of glucocorticoids may drive calcification. Due to these findings, the question of what celecoxib’s role may be in the care setting remains unclear. However, these results provide a pathway forward for research to identify either new contraindications or therapeutic uses of celecoxib and its derivative drugs in the context of AVC.

## 9. Discussion

Based on recent research findings, it is evident that the question of whether COX inhibition impacts the development of AVC, whether protective or pathogenic, requires further investigation. The only COX inhibitors that have had any recent investigation in this regard are celecoxib and its derivatives, and even that research is sparse and has conflicting results. In particular, there is a lack of studies on medical patients investigating this potential association between the COX pathways and AVC, with the exception of two retrospective clinical analyses with conflicting results [19,22]. There is a need for further retrospective studies of subjects taking COX inhibitors and simultaneously being monitored for the progression of aortic stenosis.

More research is also needed to clarify the role that celecoxib plays in AVC development in a manner that does not involve COX-2 inhibition. Since it is likely that there are other off-target pathways playing a role in celecoxib’s effect on AVC, in order to determine the possibility of the COX pathways themselves having an impact on AVC, it may be necessary for other COX inhibitors to be studied as well in this regard. Despite the lack of knowledge about COX inhibition’s role in the development of AVC, celecoxib clearly has a potential glucocorticoid-dependent effect that increases the risk of AVC development. Further investigation is needed as to whether this means the administration of celecoxib or other COX inhibitors may lead to an increased risk for the development of aortic stenosis in an in vivo environment with exposure to serum glucocorticoids.

A potentially confounding variable influencing the results of studies with celecoxib is the CYP2C9*3 polymorphism that is found fairly frequently in Caucasian populations. CYP2C9 is a polymorphic enzyme involved in the metabolism of drugs such as NSAIDs, phenytoin, and (S)-warfarin, among others [27]. The genetic polymorphism CYP2C9*3 has been shown to lead to a statistically significant reduction in CYP2C9 activity of up to five- to ten-fold in homozygous carriers in in vitro studies [28]. Furthermore, there was more than a two-fold reduction in the oral clearance of celecoxib for homozygotes for CYP2C9*3 when compared to the wild type and heterozygotes [28], suggesting that patients with these mutations could be at risk for increased dose-related effects of celecoxib.

Notably, certain conclusions in this report are based on results from only one or two studies with varying methods, including studies that had findings directly contradictory to one another. These limitations, including the lack of available studies and inconsistency in study types, must be acknowledged. Although this report provides an overview of celecoxib and its implications in CAVD, it underscores the importance of further research to replicate these findings, given the significant clinical implications of potential pharmacotherapeutics for the prevention of CAVD.

## 10. Conclusions

As of the date of this review, aortic stenosis due to age-related wear and tear has no effective pharmacotherapy in widespread use. The progression of AVC to aortic stenosis necessitates surgical intervention; therefore, investigating potential therapeutics is highly important. Based on recent studies of celecoxib and its in vitro effects on the calcification of aortic valve leaflets, it is possible that it may have a mechanism of action, either due to a CDH11 blockade or through another mechanism, that could fulfill this need. As a result, more studies to identify the connection between celecoxib and AVC are needed for the dual purpose of better informing the current use of this drug and other COX-2 inhibitors, as well as identifying possibilities for potential pharmacotherapeutic intervention. Conversely, the possibility of celecoxib or other COX inhibitors’ involvement in driving a pathogenic process leading to AVC and subsequent aortic stenosis should also be thoroughly investigated.

## Figures and Tables

**Table 1:** A summary of the major findings of recent primary studies that investigated the potential association between celecoxib derivatives and the development of aortic valve calcification [18,19,21,22,23].

Study	Proposed Effect of COX-2 on CAVD Development	Proposed Effect of Celecoxib Derivatives on CAVD Development	Proposed Pathomechanism	Study Type
Delaney et al. [22]	No association	No association	_	Human retrospective study
Wirrig et al. [18]	COX-2 expression is increased in calcified aortic valves	Reduced development of calcification	COX-2 expression leads to increased osteogenic activity	Animal and human study-in vivo and in vitro
Vieceli Dalla Sega et al. [21]	COX-2 expression is decreased in calcified aortic valves	Increased development of calcification	COX-2 inhibition induces trans-differentation of AVIC’s into myofibroblasts and increased expression of TGF-β	Human ex vivo study
Bowler et al. [19]	Not investigated	Celecoxib leads to increased calcification; DMC leads to decreased calcification	Myofibroblast induction increases calcification; CDH11 blockade decreases calcification	Human in vitro and retrospective study
Vaidya et al. [23]	Not investigated	Increased development of calcification in the presence of glucocorticoids	COX-2 inhibitors have an unknown glucocorticoid-dependent effect	Animal ex vivo study

## Data Availability

Data sharing is not applicable to this publication, as no new data were created for the purpose of this review.

## References

[1] Alushi B., Curini L., Christopher M.R., Grubitzch H., Landmesser U., Amedei A., Lauten A. (2020). Calcific Aortic Valve Disease-Natural History and Future Therapeutic Strategies. Front. Pharmacol..

[2] Lindman B.R., Clavel M.-A., Mathieu P., Iung B., Lancellotti P., Otto C.M., Pibarot P. (2016). Calcific aortic stenosis. Nat. Rev. Dis. Primer..

[3] Yi B., Zeng W., Lv L., Hua P. (2021). Changing epidemiology of calcific aortic valve disease: 30-year trends of incidence, prevalence, and deaths across 204 countries and territories. Aging.

[4] Pujari S.H., Agasthi P. (2021). Aortic Stenosis. StatPearls.

[5] Rajamannan N.M., Evans F.J., Aikawa E., Grande-Allen K.J., Demer L.L., Heistad D.D., Simmons C.A., Masters K.S., Mathieu P., O’Brien K.D. (2011). Calcific Aortic Valve Disease: Not Simply a Degenerative Process A Review and Agenda for Research from the National Heart and Lung and Blood Institute Aortic Stenosis Working Group. Circulation.

[6] Freeman R.V., Otto C.M. (2005). Spectrum of Calcific Aortic Valve Disease: Pathogenesis, Disease Progression, and Treatment Strategies. Circulation.

[7] Sucosky P., Balachandran K., Elhammali A., Jo H., Yoganathan A.P. (2009). Altered shear stress stimulates upregulation of endothelial VCAM-1 and ICAM-1 in a BMP-4- and TGF-beta1-dependent pathway. Arterioscler. Thromb. Vasc. Biol..

[8] Park J.Y., Pillinger M.H., Abramson S.B. (2006). Prostaglandin E2 synthesis and secretion: The role of PGE2 synthases. Clin. Immunol..

[9] Vane J.R., Botting R.M. (1998). Anti-inflammatory drugs and their mechanism of action. Inflamm. Res..

[10] Grosser T., Fries S., FitzGerald G.A. (2006). Biological basis for the cardiovascular consequences of COX-2 inhibition: Therapeutic challenges and opportunities. J. Clin. Investig..

[11] Kaden J.J., Bickelhaupt S., Grobholz R., Haase K.K., Sarιkoç A., Kιlιç R., Brueckmann M., Lang S., Zahn I., Vahl C. (2004). Receptor activator of nuclear factor kappaB ligand and osteoprotegerin regulate aortic valve calcification. J. Mol. Cell Cardiol..

[12] Weiss R.M., Lund D.D., Chu Y., Brooks R.M., Zimmerman K.A., El Accaoui R., Davis M.K., Hajj G.P., Zimmerman M.B., Heistad D.D. (2013). Osteoprotegerin Inhibits Aortic Valve Calcification and Preserves Valve Function in Hypercholesterolemic Mice. PLoS ONE.

[13] Pawade T.A., Newby D.E., Dweck M.R. (2015). Calcification in Aortic Stenosis: The Skeleton Key. J. Am. Coll. Cardiol..

[14] Fujisaka T., Hoshiga M., Hotchi J., Takeda Y., Jin D., Takai S., Hanafusa T., Ishizaka N. (2013). Angiotensin II promotes aortic valve thickening independent of elevated blood pressure in apolipoprotein-E deficient mice. Atherosclerosis.

[15] Arishiro K., Hoshiga M., Negoro N., Jin D., Takai S., Miyazaki M., Ishihara T., Hanafusa T. (2007). Angiotensin receptor-1 blocker inhibits atherosclerotic changes and endothelial disruption of the aortic valve in hypercholesterolemic rabbits. J. Am. Coll. Cardiol..

[16] Desai C.S., Roselli E.E., Svensson L.G., Bonow R.O. (2013). Transcatheter aortic valve replacement: Current status and future directions. Semin. Thorac. Cardiovasc. Surg..

[17] Otto C.M., Nishimura R.A., Bonow R.O., Carabello B.A., Erwin J.P., Gentile F., Jneid H., Krieger E.V., Mack M., McLeod C. (2021). 2020 ACC/AHA Guideline for the Management of Patients with Valvular Heart Disease: A Report of the American College of Cardiology/American Heart Association Joint Committee on Clinical Practice Guidelines. Circulation.

[18] Wirrig E.E., Gomez M.V., Hinton R.B., Yutzey K.E. (2015). COX2 Inhibition Reduces Aortic Valve Calcification In Vivo. Arterioscler Thromb. Vasc. Biol..

[19] Bowler M.A., Raddatz M.A., Johnson C.L., Lindman B.R., Merryman W.D. (2019). Celecoxib Is Associated With Dystrophic Calcification and Aortic Valve Stenosis. JACC Basic Transl. Sci..

[20] Schjerning A.M., McGettigan P., Gislason G. (2020). Cardiovascular effects and safety of (non-aspirin) NSAIDs. Nat. Rev. Cardiol..

[21] Sega F.V.D., Fortini F., Cimaglia P., Marracino L., Tonet E., Antonucci A., Moscarelli M., Campo G., Rizzo P., Ferrari R. (2020). COX-2 Is Downregulated in Human Stenotic Aortic Valves and Its Inhibition Promotes Dystrophic Calcification. Int. J. Mol. Sci..

[22] Delaney J.A., Lehmann N., Jöckel K.-H., Elmariah S., Psaty B.M., Mahabadi A.A., Budoff M., Kronmal R.A., Nasir K., O’Brien K.D. (2013). Associations between Aspirin and other non-steroidal anti-inflammatory drugs and aortic valve or coronary artery calcification: The Multi-Ethnic Study of Atherosclerosis and the Heinz Nixdorf Recall Study. Atherosclerosis.

[23] Vaidya K.A., Donnelly M.P., Gee T.W., Ibrahim Aibo M.A., Byers S., Butcher J.T. (2020). Induction of aortic valve calcification by celecoxib and its COX-2 independent derivatives is glucocorticoid-dependent. Cardiovasc. Pathol..

[24] Xie C., Ming X., Wang Q., Schwarz E.M., Guldberg R.E., O’Keefe R.J., Zhang X. (2008). COX-2 from the injury milieu is critical for the initiation of periosteal progenitor cell mediated bone healing. Bone.

[25] Howes L.G. (2007). Selective COX-2 inhibitors, NSAIDs and cardiovascular events—Is celecoxib the safest choice?. Ther. Clin. Risk Manag..

[26] Hutcheson J.D., Chen J., Sewell-Loftin M.K., Ryzhova L.M., Fisher C.I., Su Y.R., Merryman W.D. (2013). Cadherin-11 regulates cell-cell tension necessary for calcific nodule formation by valvular myofibroblasts. Arterioscler. Thromb. Vasc. Biol..

[27] Wanounou M., Shaul C., Abu Ghosh Z., Alamia S., Caraco Y. (2022). The Impact of CYP2C9*11 Allelic Variant on the Pharmacokinetics of Phenytoin and (S)-Warfarin. Clin. Pharmacol. Ther..

[28] Kirchheiner J., Störmer E., Meisel C., Steinbach N., Roots I., Brockmöller J. (2003). Influence of CYP2C9 genetic polymorphisms on pharmacokinetics of celecoxib and its metabolites. Pharmacogenetics.

